# Self-critical perfectionism mediates the relationship between self-esteem and satisfaction with life in Lebanese university students

**DOI:** 10.1186/s40359-023-01040-6

**Published:** 2023-01-07

**Authors:** Feten Fekih-Romdhane, Toni Sawma, Sahar Obeid, Souheil Hallit

**Affiliations:** 1 The Tunisian Center of Early Intervention in Psychosis, Department of Psychiatry “Ibn Omrane”, Razi Hospital, 2010 Manouba, Tunisia; 2 Faculty of Medicine of Tunis, Tunis El Manar University, Tunis, Tunisia; 3 Social and Education Sciences Department, School of Arts and Sciences, Lebanese American University, Jbeil, Lebanon; 4 School of Medicine and Medical Sciences, Holy Spirit University of Kaslik, P.O. Box 446, Jounieh, Lebanon; 5 Research Department, Psychiatric Hospital of the Cross, Jal Eddib, Lebanon; 6 Applied Science Research Center, Applied Science Private University, Amman, Jordan

**Keywords:** Perfectionism, Self-esteem, Satisfaction with life, Students

## Abstract

**Background:**

The psychological mechanisms that underlie the relationship between self-esteem and life satisfaction remain unclear and not well-understood. We sought through the present study to test the hypothesis that perfectionism plays a significant mediating role in the association between self-esteem and satisfaction with life among Lebanese university students.

**Methods:**

A cross-sectional study was performed in a sample of 363 students (61.7% females, mean age = 22.65 ± 3.48 years). Three research instruments were used: satisfaction with life scale, Rosenberg self-esteem scale and big three perfectionism scale.

**Results:**

Higher self-esteem was significantly associated with lower self-critical perfectionism (Beta = − 0.47), whereas higher self-critical perfectionism was significantly associated with lower satisfaction in life (Beta = − 0.29). Finally, higher self-esteem was significantly and directly associated with higher satisfaction with life (Beta = 0.48).

**Conclusion:**

The present preliminary findings point to the role of self-critical perfectionism as a buffer between self-esteem and satisfaction with life, suggesting the roles of self-esteem and perfectionism as promising avenues for promoting satisfaction with life in adolescent students.

## Background

Self-esteem (SE) is a personality variable that refers to one’s self-related feelings and overall self-assessment that is either positive (the person believes they are valued by others and “good enough”), or negative (the person suffers self-rejection or is dissatisfied with themselves) [1–3]. SE trait was found to be inversely associated with various negative mental health outcomes, including higher levels of depression [4], anxiety, social phobia [5], social media addiction [6], loneliness [7], more negative social relationships, and less social support over time [8]. On another hand, high SE is positively associated with a range of positive and adaptive outcomes [9], such as higher happiness [10] and more positive life satisfaction [6, 11].

SE effects are particularly impactful during late adolescence and early adulthood, where individuals are at an age of acquiring knowledge, developing of new competences, and expanding social roles; but also at a peak age of onset for mental disorders [12]. In this age range, students represent a vulnerable group for both SE issues and mental health problems, as they are exposed to different kinds of physical and psychological stressors and high stress levels [13]. SE was found to be strongly related to academic achievement [14], mental health [15], and to positively predict life satisfaction [16–20] in university students. In addition, SE has been found to be significantly and positively correlated with general self-efficacy among elite university students [21], as well as other students enrolled in different fields of specialization [22]. Interestingly, a recent Chinese longitudinal study has also found that SE levels declined over years, and has negative prospective impacts on depression among university students [23]. Another large longitudinal study demonstrated that SE maintained having substantial and durable negative impacts on anxiety levels among university students over time [24]. SE seems thus to play a crucial role in causing and maintaining psychopathology in students, and should be given full consideration when aiming to promote mental health in this population.

Satisfaction with life represents a part of the subjective well-being in one's life [25]. Nowadays, efforts promoting positive psychology and overall well-being among adolescents is becoming of great significance to public health and society [26]. There is sufficient available evidence to support the positive significant effect between SE and life satisfaction [27]. A strong evidence has been accumulated on the positive cross-sectional and longitudinal link between SE and life satisfaction across gender and cultures [28–30]. Theorists suggest different frameworks to emphasize that Satisfaction with life could be an outcome variable of SE. For instance, Diener and Diener suggested that people from individualist societies tend to evaluate their lives mainly based on internal rather than external personal attributes, including SE [30]. Indeed, consistent with the self-construal theory [31, 32], people (individualists more than collectivists [30], and males more than females [33, 34]) would put heavier weight on internal sources (such as SE) when evaluating their lives.

However, several key knowledge gaps in the existing literature could be identified. First, prior research produced mixed results, revealing a positive association between SE and life satisfaction in some studies (e.g., [11, 35]) and a negative association in others (e.g., [36]); and indicating a need to further clarify this relationship. Second, both SE and satisfaction with life vary across countries [20]. Cross-cultural and cross-country differences in the strength of associations between SE and life satisfaction have also been observed in previous studies; depending on whether societies are individualistic or collectivist in nature [30]. However, most previous studies have been conducted in Western and individualistic settings (European countries and the United States). It remains thus unclear whether the influence of SE on life satisfaction can apply to student populations from under-researched countries such as Lebanon that has a collectivist Arab culture. Third, although the improvement in SE and subsequent life satisfaction has been considered as a central preoccupation over the last decades in Western countries [37], available intervention strategies aiming at boosting SE demonstrated poor effectiveness (e.g., [38, 39]) suggesting that alternative therapies need to be developed. This should start by investigating why individuals having poor SE experience low levels of life satisfaction.

Nevertheless, examining the relationship SE-life satisfaction is challenging, since both concepts are multifaceted and complex constructs [40, 41], and both are subjective in nature, based on personal assessment and perception. A number of mediators of the association between SE and satisfaction with life have been examined in the search for an understanding of the patterns of this relationship, such as age [42], gender [35, 43], socioeconomic status [28, 44], parenting styles [45], internet usage [46, 47], health conditions [43], and social status [42]. Notwithstanding these studies’ findings, the psychological mechanisms that underlie the relationship SE and satisfaction with life remain unclear and not well-understood. One potential mediator that has never previously been examined, and that we propose to explore in this study is perfectionism.

Perfectionism can be defined as being overly self-critical, and setting very high behavioral and personal performance norms [48]. Many university students experience prominent academic pressures and success expectations; consequently, perfectionism is found to be prevalent in this population [49, 50]. Students with perfectionistic tendencies are at increased risk of mental-health issues [51], and are assumed to have decreased SE levels [52]. Early and more recent theoretical and empirical work highlighted the determinant role of SE for the prediction of personality development, and more particularly the development of perfectionism [53, 54]. Also, perfectionist students, particularly those exhibiting high levels of maladaptive perfectionism, are shown to have low level of life satisfaction (e.g., [55, 56]). Indeed, adaptive and maladaptive perfectionism have been suggested as being, respectively, an enhancement or an impairment to overall life satisfaction [57]. Therefore, perfectionism would either lead one to live a highly satisfying life (when adaptive) [49, 58], or have unhealthy physical and psychological effects (when maladaptive) (e.g., [57, 59]).

To our knowledge, there have been no previous studies, so far, that have explored how perfectionist traits (narcissistic, rigid, and self-critical) may provide an explanatory pathway between SE and life satisfactions. Gaining more knowledge on the relationship between SE and life satisfaction may be informative for school-based psychological interventions aimed at increasing SE and promoting the well-being of students [43, 60]. In other words, if perfectionism is found to mediate the effects of SE on life satisfaction, then perfectionism could be a mendable target for low SE and life satisfaction treatment. In this context, we sought through the present study to test the hypothesis that perfectionism plays a significant mediating role in the association between SE and satisfaction with life among Lebanese university students. Therefore, we suggest that a lower SE could negatively affect satisfaction with life via a high perfectionism. The intended target audience of this paper are clinicians, educators, administrators, all school practitioners who work with students, as well as educational policymakers seeking to promote students' mental health and well-being.

## Methods

### Study design and participants

This cross-sectional study was carried out between July and September 2021. A total of 363 university students were recruited through convenience sampling through several universities in Lebanon’s governorates. Participants received the online link to the survey. Involved people were encouraged to visit a website that would guide them to the consent form, information form (purpose of the current study, anonymity, voluntariness of consent to research), and questionnaire. All participants responded willingly to the survey. There were no fees for participating in the study. All university students over the age of 18 were eligible to participate. Excluded were those who refused to complete the survey [61, 62].

### Minimal sample size calculation

According to the G-power, a minimum of 316 students was deemed necessary to have enough statistical power, based on a 5% risk of error, 80% power, f^2^ = 2.5% and 10 factors to be entered in the multivariable analysis.

### Questionnaire and variables

The Arabic self-administered questionnaire with closed-ended questions was anonymous; the questionnaire required approximately 20 min to be completed. The questionnaire consisted of different sections. The first part clarified socio-demographic characteristics: age, gender, marital status, and household crowding index. The latter, reflecting the socioeconomic status of the family, was calculated by dividing the number of persons in the house by the number of rooms in the house excluding the bathrooms and kitchen [63]. The physical activity index was calculated by multiplying the intensity by the frequency by the time of physical activity [64].

The second part of the questionnaire included the following scales:

### Satisfaction with life scale (SWLS)

This tool is composed of 5 statements, scored on a five-point Likert scale (1 = strongly disagree to 5 = strongly agree). The SWLS is validated in Arabic [65]. Higher scores indicate more satisfaction with life (αCronbach = 0.89).

### Rosenberg Self‐Esteem Scale (RSES)

It is a 10-item scale that reflects self-worth by focusing on both positive and negative feelings people have about themselves [66]. Items are scored on a four-point Likert scale (1 = strongly disagree to 4 = strongly agree). Higher scores reflect a better SE. The Arabic version has been used in previous papers [67, 68] (αCronbach = 0.87).

### Big three perfectionism scale

This scale is composed of 16 items, scored on a five-point Likert scale (1 = strongly disagree to 5 = strongly agree). It yields three subscales scores: rigid perfectionism, self-critical perfectionism and narcissistic perfectionism. Higher scores reflect higher perfectionism in the three aspects. In this study, the Cronbach’s alpha values for the three scores were as follows: rigid perfectionism (α = 0.87), self-critical perfectionism (α = 0.88) and narcissistic perfectionism (α = 0.81).

### Statistical analysis

SPSS software version 25 was used to conduct data analysis. The normality of the satisfaction with life, SE and perfectionism subscales scores were verified via the skewness and kurtosis values varying between − 1 and + 1 [69]. A bivariate analysis using the Pearson correlation test served to assess the relationship between the satisfaction with life score and other continuous variables, whereas the Student t test was used to compare two means. The PROCESS SPSS Macro v. 3.4, Model 4 [70] was used to conduct the mediation analysis; three pathways were calculated: (a) Relation between SE and perfectionism; (b) Relation between perfectionism and satisfaction with life; (c’) Direct effect of the relation between SE and satisfaction with life. The number of bootstrap sampling used was 5000. The mediation analysis was adjusted over all sociodemographic variables that showed a *p* < 0.25 in the bivariate analysis for the elimination of confounding factors as much as possible. Significance was defined at *p* < 0.05.

## Results

### Sociodemographic and other characteristics of the participants

A total of 363 students participated in this study; their mean age was 22.65 ± 3.48 years, with 61.7% females. Other characteristics are summarized in Table 1.

**Table 1:** Sociodemographic and other characteristics of the participants (n = 363)

Variable	n (%)
Sex	
Male	139 (38.3%)
Female	224 (61.7%)
Marital status	
Single	343 (94.5%)
Married	20 (5.5%)

	Mean ± SD
Age (in years)	22.65 ± 3.48
Physical activity index	27.94 ± 20.44
Household crowding index	1.01 ± 0.53
Satisfaction with life score	23.05 ± 6.45
Rigid perfectionism score	12.61 ± 3.80
Self-critical perfectionism score	17.60 ± 5.63
Narcissistic perfectionism score	14.29 ± 4.76
Self-esteem score	16.14 ± 2.09

### Bivariate analysis

The bivariate analysis results are shown in Tables 2 and 3. Higher SE (r = 0.41) was significantly associated with a higher satisfaction with life, whereas higher rigid perfectionism (r = − 0.24) and self-critical perfectionism (r = − 0.39) were significantly associated with lower satisfaction with life.

**Table 2:** Bivariate analysis of the categorical variables associated with satisfaction with life

Variable	Satisfaction with life		Rigid perfectionism		Self-critical perfectionism		Narcissistic perfectionism		Self-esteem	
	Mean ± SD	*p*	Mean ± SD	*p*	Mean ± SD	*p*	Mean ± SD	*p*	Mean ± SD	*p*
Sex		0.673		0.670		0.091		0.244		0.844
Male	22.87 ± 6.66		12.50 ± 3.66		16.96 ± 5.42		14.66 ± 4.69		20.78 ± 5.06	
Female	6.34 ± 0.42		12.68 ± 3.89		17.99 ± 5.73		14.06 ± 4.80		20.89 ± 5.53

**Table 3:** Bivariate analysis of the continuous variables associated with satisfaction with life

Variable	1	2	3	4	5	6	7	8	9
1. Satisfaction with life	1								
2. Age	0.10	1							
3. Physical activity index	− 0.01	− 0.13*	1						
4. Household crowding index	− 0.09	− 0.12*	0.02	1					
5. Body Mass Index	− 0.01	− 0.29***	− 0.08	0.02	1				
6. Rigid perfectionism	− 0.24***	0.02	0.02	− 0.04	− 0.10	1			
7. Self-critical perfectionism	− 0.39***	− 0.08	− 0.09	0.07	− 0.11*	0.53***	1		
8. Narcissistic perfectionism	− 0.09	0.01	− 0.05	− 0.002	− 0.05	0.49***	0.51***	1	
9. Self-esteem	0.41***	0.11*	0.04	− 0.09	− 0.02	− 0.10	− 0.46***	− 0.26***	1

Numbers in the table refer to Pearson correlation coefficients; *p < 0.05; ***p < 0.001

### Mediation analysis

**Fig. 1:**
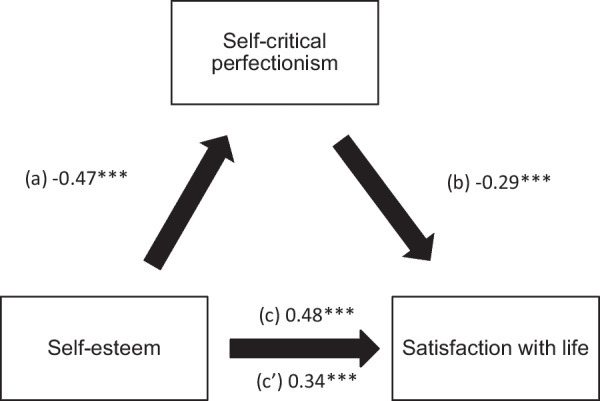
**a** Relation between self-esteem and self-critical perfectionism (R^2^ = 0.21); **b** Relation between self-critical perfectionism and satisfaction with life (R^2^ = 0.22); **c** Total effect of the relation between self-esteem and satisfaction with life (R^2^ = 0.17); **c’** Direct effect of the relation between self-esteem and satisfaction with life. Numbers are displayed as regression coefficients. ***p < 0.001

The results of the mediation analysis showed that self-critical perfectionism mediated the association between SE and satisfaction with life (Table 4 and Fig. 1). Higher self-esteem was significantly associated with lower self-critical perfectionism (Beta = − 0.47), whereas higher self-critical perfectionism was significantly associated with lower satisfaction in life (Beta = − 0.29). Finally, higher self-esteem was significantly and directly associated with higher satisfaction with life (Beta = 0.48).

**Table 4:** Mediation analyses results, taking self-esteem as the independent variable, the perfectionism subscales as mediators and satisfaction with life as the dependent variable

	Direct effect			Indirect effect		
	Beta	SE	*p*	Beta	Boot SE	Boot CI
Rigid perfectionism	0.45	0.06	< 0.001	0.03	0.02	− 0.002; 0.06
Self-critical perfectionism	0.34	0.06	< 0.001	0.14	0.04	0.07; 0.21*
Narcissistic perfectionism	0.49	0.06	< 0.001	−0.001	0.02	− 0.04; 0.03

*Indicates significant mediation

## Discussion

In the present study, we examined the mediating effects of the different perfectionistic components or facets in the relationship between SE and life satisfaction. The study rationale is to suggest possible strategies for alleviating students’ SE and satisfaction with life issues through addressing a modifiable factor, high perfectionism. As expected, mediation analysis found that one particular dimension of perfectionism, self-critical perfectionism, played a significant indirect effect on the relationship SE – satisfaction with life among Lebanese university students.

The first finding of the present study supports earlier studies indicating that lower SE as well as higher rigid and self-critical perfectionism were significantly associated with lower life satisfaction. In agreement with our findings, several international studies from India [17], China [71], Poland, Spain, Slovakia [20], Italy and the US [47] documented a significant effect of SE on satisfaction with life among students. This means that students having poor SE were more likely to experience decreased satisfaction with life. Concerning the relationship between perfectionism and life satisfaction, previous studies documented differential effects according to perfectionism dimensions. For instance, in line with our findings, a Turkish study reported a negative correlation between maladaptive perfectionism and life satisfaction among high-school students [72]. Previous studies have shown that some maladaptive perfectionism traits, such as self-critical perfectionism, are rigid cognitive processes that have been found to be significant predictors of psychological distress which in turn lead to reduced life satisfaction [73]. Also, perfectionism is a stressful mode of coping that is likely to result in shame and self-devaluation after failure [74]. Other studies highlighted a lack of association between adaptive perfectionism and life-satisfaction among Turkish [75], Canadian [73], Chinese [76], English [77], and black American [78] university students. In sum, even though the available literature data revealed some inconsistencies, the different perfectionism dimensions showed to be often moderately correlated with each other’s, but to be linked to differing outcomes (e.g., [79–81]).

This latter claim is also congruent with the mediation analysis finding, which revealed that only self-critical perfectionism had a significant mediating effect on the relationship between SE and life satisfaction. These findings suggest that lower SE can lead to an overall poor sense of satisfaction with life when students have higher levels of self-critical perfectionism. This means that SE is a personality variable which not only directly contributed to students’ life satisfaction, but also indirectly through high self-critical perfectionism levels. Consistent with our findings, a previous Australian study performed among medical students found that maladaptive perfectionistic tendencies act as moderating factors on the association between some personality trait profiles (i.e. harm avoidance, high cooperation, self-directedness and persistence) and levels of depression, anxiety and stress [82]. Our results are also broadly consistent with earlier investigations which showed that perfectionism mediates the relationship between SE and negative outcomes such as work craving [83]. On the other hand, neither narcissistic nor rigid perfectionism had a significant mediating role in the relationship between SE and life satisfaction. Higher scores were obtained for the Self-critical perfectionism subscale, which might suggest why this perfectionism facet has been more impactful as a mediator than others. However, as this is the first attempt to explore the effect of perfectionism dimensions in this relationship, we call for future longitudinal research to further investigate the nature of the associations found in the present study. Overall, the present findings suggest that interventions aiming at decreasing self-critical perfectionism could possibly prevent low SE from resulting in psychopathology.

### Study strengths and limitations

This study has several strengths. First, we focused on students’ positive psychological outcomes that are gaining a growing interest in school settings, while most of the previous studies only investigated negative psychological outcomes in relation to SE and perfectionism (e.g., eating disorders, social anxiety, stress, and depression). Second, we used the Big Three Perfectionism Scale to examine students’ perfectionism traits, which allowed for the assessment of three perfectionism factors (rigid, self- critical, and narcissistic), in contrast to previous studies where perfectionism was not subdivided. Our approach offers thus an in-depth exploration of the relationship between perfectionism and psychological outcomes. Third, this is one of the limited studies that involved participants from a collectivist country and an under-explored region.

Notwithstanding these strengths, this study has limitations that need to be considered. First, the cross-sectional design precludes from determining the direction of relationships. Second, we used self-reported method, which cannot prevent response bias due to social desirability [84]. Third, other psychological factors (such as depression, loneliness and social support) may also account for aspects of the associations between the studied variables predisposing us to a residual confounding bias, and their role should be considered in future investigations. A selection bias is present because of the sampling method and the refusal rate. Finally, there is a lack of diversity of the sample in terms of gender or marital status, which may have had some influence on the obtained results.

### Clinical and research implications

Although our findings are preliminary and require further confirmation on longitudinal studies, a few implications can be drawn for practice and theory. Mediation analysis showed that low SE can elicit less positive life satisfaction when people tend to be self-critical perfectionists. This finding point to the potential value for school practitioners who work with students having low SE to take perfectionism into account when aiming to enhance students’ satisfaction with life and overall wellbeing. School-based interventions focusing on ways to decrease students’ levels of maladaptive perfectionists may be effective in increasing their life-satisfaction levels [82]. In medical schools, where students are typically known to be highly competitive, high achievers and perfectionists, such interventions aiming at improving students’ mental health revealed encouraging results [85–88].

Another approach that has been proposed is early screening of students for dysfunctional perfectionism [88]. Students with low SE are likely to be highly self-critical perfectionists, and could be targeted by screening and interventions. However, when identifying students most in need of interventions, caution is required regarding the possibility of stigmatization and labeling [89].

School counselors are also recommended to raise awareness among students about the potential harmful effects of maladaptive perfectionism (e.g., comparisons to others, high competitiveness, fear of judgment, excessive self-doubt) on personal well-being and mental health outcomes. Some researchers even suggested to incorporate these awareness programs into the curriculum (e.g., [82]). It is worth noting, however, that perfectionism has proven to have both positive and negative impact on students’ psychological outcomes [79]. It remains thus challenging to understand and determine when perfectionism should be considered a target strategy to reduce vulnerability of students to poor mental health outcomes, versus when it could be accepted and even embraced. This calls for additional studies focusing on perfectionism when studying students’ psychological life satisfaction.

## Conclusion

The findings of this paper support and add to the existing evidence, that has mainly emerged from Asian (Collectivist) and Western (Individualist) countries, on the positive association between SE and life satisfaction; by examining this relationship for the first time in a Middle East country. Also, the current study is unique as it enhances our understanding of the mediatory role of perfectionism in the relationship SE – satisfaction with life. There are no previous studies that have looked at the mediating effect of three dimensions of perfectionism (narcissistic, rigid, and self-critical) in this relationship. The findings point to the role of self-critical perfectionism as a buffer between SE and satisfaction with life, suggesting the roles of SE and perfectionism as promising avenues for promoting satisfaction with life in adolescent students. Future studies in various populations, settings and countries are needed to confirm and extend these findings.

## Data Availability

All data generated or analyzed during this study are not publicly available to maintain the privacy of the individuals’ identities. The dataset supporting the conclusions is available upon request to the corresponding author.
